# Identification of a Spike-Specific CD8^+^ T-Cell Epitope Following Vaccination Against the Middle East Respiratory Syndrome Coronavirus in Humans

**DOI:** 10.1093/infdis/jiad612

**Published:** 2024-01-09

**Authors:** Caroline E Harrer, Leonie Mayer, Anahita Fathi, Susan Lassen, My L Ly, Madeleine E Zinser, Timo Wolf, Stephan Becker, Gerd Sutter, Christine Dahlke, Marylyn M Addo, for the MVA-MERS-S Study Group, Etienne Bartels, Monika Friedrich, Leonie M Weskamm, Swantje Grundlach, Joseph H Poetsch, Till Koch, Stefan Schmiedel, Bart Haagmanns, Thomas Hesterkamp, Verena Krähling, Asisa Volz, Vanessa A Ditt, Melanie Kessler

**Affiliations:** Institute for Infection Research and Vaccine Development, University Medical Center Hamburg-Eppendorf; Department for Clinical Immunology of Infectious Diseases, Bernhard Nocht Institute for Tropical Medicine; German Center for Infection Research, partner site Hamburg-Lübeck-Borstel-Riems, Hamburg; Institute for Infection Research and Vaccine Development, University Medical Center Hamburg-Eppendorf; Department for Clinical Immunology of Infectious Diseases, Bernhard Nocht Institute for Tropical Medicine; German Center for Infection Research, partner site Hamburg-Lübeck-Borstel-Riems, Hamburg; Institute for Infection Research and Vaccine Development, University Medical Center Hamburg-Eppendorf; Department for Clinical Immunology of Infectious Diseases, Bernhard Nocht Institute for Tropical Medicine; German Center for Infection Research, partner site Hamburg-Lübeck-Borstel-Riems, Hamburg; First Department of Medicine, Division of Infectious Diseases, University Medical Center Hamburg-Eppendorf, Hamburg; Institute for Infection Research and Vaccine Development, University Medical Center Hamburg-Eppendorf; Department for Clinical Immunology of Infectious Diseases, Bernhard Nocht Institute for Tropical Medicine; German Center for Infection Research, partner site Hamburg-Lübeck-Borstel-Riems, Hamburg; Institute for Infection Research and Vaccine Development, University Medical Center Hamburg-Eppendorf; Department for Clinical Immunology of Infectious Diseases, Bernhard Nocht Institute for Tropical Medicine; German Center for Infection Research, partner site Hamburg-Lübeck-Borstel-Riems, Hamburg; Institute for Infection Research and Vaccine Development, University Medical Center Hamburg-Eppendorf; Department for Clinical Immunology of Infectious Diseases, Bernhard Nocht Institute for Tropical Medicine; German Center for Infection Research, partner site Hamburg-Lübeck-Borstel-Riems, Hamburg; Goethe University Frankfurt, University Hospital, Department of Internal Medicine II, Division of Infectious Diseases, Frankfurt am Main; German Center for Infection Research, partner site Gießen-Marburg-Langen, Marburg; Institute of Virology, Philipps University Marburg, Marburg; German Center for Infection Research, partner site Munich, Munich; Division of Virology, Institute for Infectious Diseases and Zoonoses, Ludwig Maximilian University of Munich, Munich, Germany; Institute for Infection Research and Vaccine Development, University Medical Center Hamburg-Eppendorf; Department for Clinical Immunology of Infectious Diseases, Bernhard Nocht Institute for Tropical Medicine; German Center for Infection Research, partner site Hamburg-Lübeck-Borstel-Riems, Hamburg; Institute for Infection Research and Vaccine Development, University Medical Center Hamburg-Eppendorf; Department for Clinical Immunology of Infectious Diseases, Bernhard Nocht Institute for Tropical Medicine; German Center for Infection Research, partner site Hamburg-Lübeck-Borstel-Riems, Hamburg

**Keywords:** MERS-CoV, epitope, CD8^+^ T cells, MVA, viral vector, vaccine

## Abstract

Licensed vaccines against the Middle East respiratory syndrome coronavirus (MERS-CoV), an emerging pathogen of concern, are lacking. The modified vaccinia virus Ankara vector-based vaccine MVA-MERS-S, expressing the MERS-CoV-spike glycoprotein (MERS-S), is one of 3 candidate vaccines in clinical development and elicits robust humoral and cellular immunity. Here, we identified for the first time a MERS-S–specific CD8^+^ T-cell epitope in an HLA-A*03:01/HLA-B*35:01-positive vaccinee using a screening assay, intracellular cytokine staining, and in silico epitope prediction. As evidence from MERS-CoV infection suggests a protective role of long-lasting CD8^+^ T-cell responses, the identification of epitopes will facilitate longitudinal analyses of vaccine-induced T-cell immunity.

## Teaser

This study reports for the first time the identification of a Middle East respiratory syndrome coronavirus (MERS-CoV) spike-specific CD8^+^ T-cell epitope in an HLA-A*03/HLA-B*35-positive individual following MVA-MERS-S vaccination. This contributes to the limited understanding of T-cell immunity against MERS-CoV.

The Middle East respiratory syndrome coronavirus (MERS-CoV) is an emerging zoonotic pathogen. It belongs to the *Betacoronavirus* genus, which includes the endemic human coronaviruses (HCoV-OC43 and HCoV-HKU1) and the epidemic SARS-CoV and SARS-CoV-2 [1, 2]. Infection with MERS-CoV leads to MERS, a primarily respiratory disease with a case fatality rate of approximately 35% [1, 2]. Since 2012, >2000 cases have been reported, mostly on the Arabian Peninsula [1, 2]. Dromedary camels serve as a natural reservoir for MERS-CoV, harboring a constant risk of spillover to humans and viral evolution [1, 2]. Due to this high epidemic potential, MERS-CoV is listed by the World Health Organization as a priority pathogen of concern for epidemic preparedness measures, such as vaccine development [1, 2].

The current vaccine candidates are predominantly based on the MERS-CoV spike protein (MERS-S), a highly immunogenic surface protein, that facilitates the binding (S1 subunit) and fusion (S2 subunit) of MERS-CoV with its host cells [2]. One of the 3 MERS vaccine candidates in clinical development is the viral vector vaccine MVA-MERS-S, based on the modified vaccinia virus Ankara (MVA) expressing the full-length MERS-S [3]. In a first-in-human phase 1 clinical trial (ClinicalTrials.gov identifier NCT03615911), we previously demonstrated the induction of robust humoral and cellular immunity after 2 MVA-MERS-S vaccinations. MERS-S–specific T-cell responses were detectable in 87% of vaccinees (n = 20/23) upon stimulation with MERS-S–specific overlapping peptide (OLP) pools [4]. Interestingly, in some vaccinees T-cell responses were already measurable after the first dose and persisted for a longer time compared to immunoglobulin G levels [4].

There is still a limited understanding of T-cell immunity elicited by MERS-CoV infection or vaccination, but evidence from MERS survivor studies suggests a protective and long-lasting role [5–7]. In particular, CD8^+^ T cells were detectable even in patients with mild or asymptomatic disease, in the absence of relevant antibody titers [5]. MERS-specific CD8^+^ T cells may therefore be important for monitoring immunogenicity of MERS vaccines longitudinally. MERS-CoV–specific T-cell epitopes, however, remain mostly unknown, hindering the development of tools, such as tetramers, for the detection, isolation, and characterization of antigen-specific T cells. While several MERS-S–specific CD4^+^ T-cell epitopes have been described in MERS survivors [5], MERS-S–specific CD8^+^ T-cell epitopes have only been predicted in silico [8] and to date have not been identified in humans.

To address these constraints, the aim of this study was to identify MERS-S–specific CD8^+^ T-cell epitopes after vaccination with the viral vector vaccine candidate MVA-MERS-S. We analyzed the T-cell response to single overlapping peptides covering MERS-S in the vaccinee with the highest overall T-cell response in the phase 1 clinical study and identified the first MERS-S–specific CD8^+^ T-cell epitope candidate after MERS vaccination.

## METHODS

The vaccinee described here was part of a first-in-human phase 1 clinical trial of the vaccine candidate MVA-MERS-S (NCT03615911, published [4]). The trial consisted of a homologous prime-boost scheme of 1 × 10^7^ or 1 × 10^8^ plaque-forming units/mL of MVA-MERS-S on days 0 and 28. The study was approved by the competent national authority (Paul Ehrlich Institute) and the Ethics Committee of the Hamburg Medical Association. Informed consent was obtained from all participants. The study was performed in accordance with the Declaration of Helsinki (2013). For immunogenicity analysis, peripheral blood mononuclear cells (PBMCs) were collected on days 7, 14, and 28 after the second vaccination (V2D7, V2D14, and V2D28, respectively). HLA typing was performed using commercially available A and B locus kits (LABType SSO Typing Test, ONE LAMDA) according to the manufacturer’s instructions.

MERS-S–specific T-cell responses were assessed using the CTL human interferon-gamma (IFN-γ) single-color 384-well enzyme-linked immunospot (ELISpot) assay (CTL ImmunoSpot). After thawing and overnight resting, 5 × 10^4^ PBMCs were stimulated for 16 hours at 37°C and 5% carbon dioxide. For stimulation, OLP pools M1–M5 (15-mers overlapping by 11 amino acids, JPT), spanning the entire MERS-S sequence (GenBank: JX869059), and single peptides (P1–P65) of pool M1 (Supplementary Table 1) were used at a peptide concentration of 1 μg/mL. Phytohemagglutinin (Sigma-Aldrich) and a cytomegalovirus–Epstein-Barr–influenza virus pool (JPT) served as positive controls, and dimethyl sulfoxide (DMSO) served as a negative control. Spot-forming cells (SFC) per million PBMCs were counted using an AID ELISpot Reader System (AID GmbH), and the results are reported as the background (DMSO)–subtracted mean count of triplicate wells.

Cytokine secretion was analyzed by intracellular cytokine staining (ICS). PBMCs were stimulated with OLP pools M1–M5 (1 μg/mL) at 37°C and 5% carbon dioxide for 7 hours in the presence of Golgi-Stop (BD Biosciences), anti-CD28/CD49 (BD Biosciences), anti-CD107a (BioLegend), and Golgi-Plug (after 2 hours of stimulation; BD Biosciences). DMSO and phorbol-12-myristate-13-acetate (50 ng/mL) plus ionomycin (0.5 μg/mL) served as negative and positive controls, respectively. PBMCs were stained with surface markers anti-CD3-BUV395 (BD Biosciences), anti-CD4-AF700, anti-CD19-BV510, anti-CD14-BV510, anti-CD8-APC-Cy7, anti-CCR7, anti-CD45RO-FITC, and Zombie Aqua Fixable Viability Kit (BioLegend) in FACS buffer (phosphate-buffered saline supplemented with 2% fetal bovine serum and 2 mM ethylenediaminetetraacetic acid). After fixation (eBioscience), PBMCs were stained with intracellular markers anti-IFN-γ-PE-Cy7, anti-tumor necrosis factor alpha (TNF-α)–PE/Dazzle 594, and anti-interleukin 2 (IL-2)–PerCP-Cy5.5 (BioLegend) in PERM buffer (eBioscience). Samples were measured on a BD Fortessa and analyzed using FlowJo software (v.10.8.1) as shown in Supplementary Figure 1. Memory T cells were identified by excluding CCR7^+^/CD45RO^−^ naive T cells. The data were normalized to the negative control. Polyfunctionality was assessed by Boolean gating.

Multiple sequence alignment of the spike protein of human betacoronaviruses was performed using the Clustal Omega web server of the European Bioinformatics Institute. Spike sequences were downloaded from the National Center for Biotechnology Information (NCBI): MERS-CoV (GenBank: JX869059), SARS-CoV-2 Wuhan (GenBank: MN908947.3), SARS-CoV BJ01 (GenBank: AY278488.2), HCoV-OC43 (NCBI reference sequence: NC_006213.1), and HCoV-HKU1 (GenBank: KF686346.1). Epitope prediction for the HLA-A*03:01 and HLA-B*35:01 alleles was performed using the NetMHCpan-4.1 software.

## RESULTS

**Figure 1.**
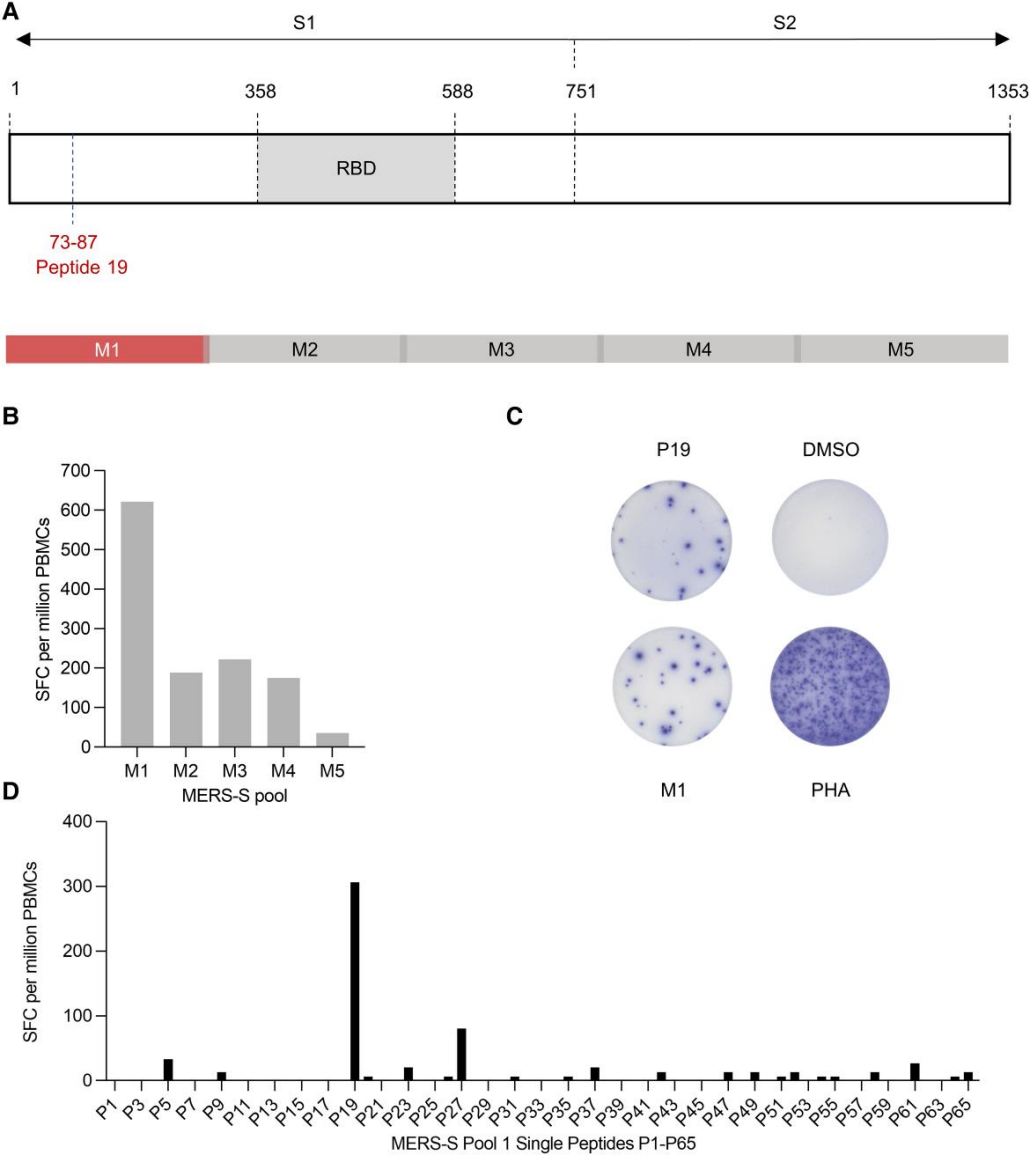
T-cell epitope mapping of the Middle East respiratory syndrome coronavirus spike protein (MERS-S) using an interferon gamma (IFN–γ) enzyme-linked immunospot (ELISpot) assay. *A*, Schematic sequence of the 1353aa spanning MERS-S protein (GenBank: JX869059) (top) and location of overlapping peptide pools M1–M5 (bottom) [9]. Peptide 19 (GLFPYQGDHGDMYVY) mapped to the S1 subunit outside the receptor-binding domain at position 73–87. *B*, Frequencies of IFN–γ–producing T cells at V2D14 after restimulation with overlapping peptide pools M1–M5 (shown as SFC per million peripheral blood mononuclear cells [PBMCs] measured by IFN–γ ELISpot, mean of technical triplicates). *C*, Representative IFN-γ ELISpot assay wells of the unstimulated negative control and after restimulation with the M1 pool, P19 peptide, and positive control. *D*, Frequencies of IFN–γ–producing T cells at V2D14 after restimulation with M1 pool single peptides (P1–P65) (shown as spot-forming cells per million PBMCs measured by IFN–γ ELISpot, mean of technical triplicates). Abbreviations: DMSO, dimethyl sulfoxide; MERS-S, Middle East respiratory syndrome coronavirus spike protein; PBMCs, peripheral blood mononuclear cells; PHA, phytohemagglutinin P; RBD, receptor-binding domain; SFC, spot-forming cells.

We analyzed MERS-S–specific T-cell immunity induced by MVA-MERS-S in an HLA-A*03:01/HLA-B*35:01 homozygous vaccinee (see detailed description in Supplementary Table 2). We used 5 OLP pools (M1–M5) covering the whole MERS-S sequence, as depicted in Figure 1*A*, for restimulation and measured the T-cell response by IFN-γ ELISpot. The highest IFN-γ response was observed after stimulation with the M1 pool (622 SFC/1 × 10^6^ PBMCs) at V2D14 (Figure 1*B*) [4]. We then used single peptides P1–P65 of the M1 pool to screen for immunogenic epitopes. P19 (GLFPYQGDHGDMYVY) elicited an IFN-γ response of 307 SFC/1 × 10^6^ PBMCs, the highest in magnitude compared with the other M1 peptides (Figure 1*C* and 1*D*). P19 mapped to the N-terminal domain of the MERS-S1 subunit outside the receptor-binding domain (RBD) at position 73–87aa (Figure 1*A*).

**Figure 2.**
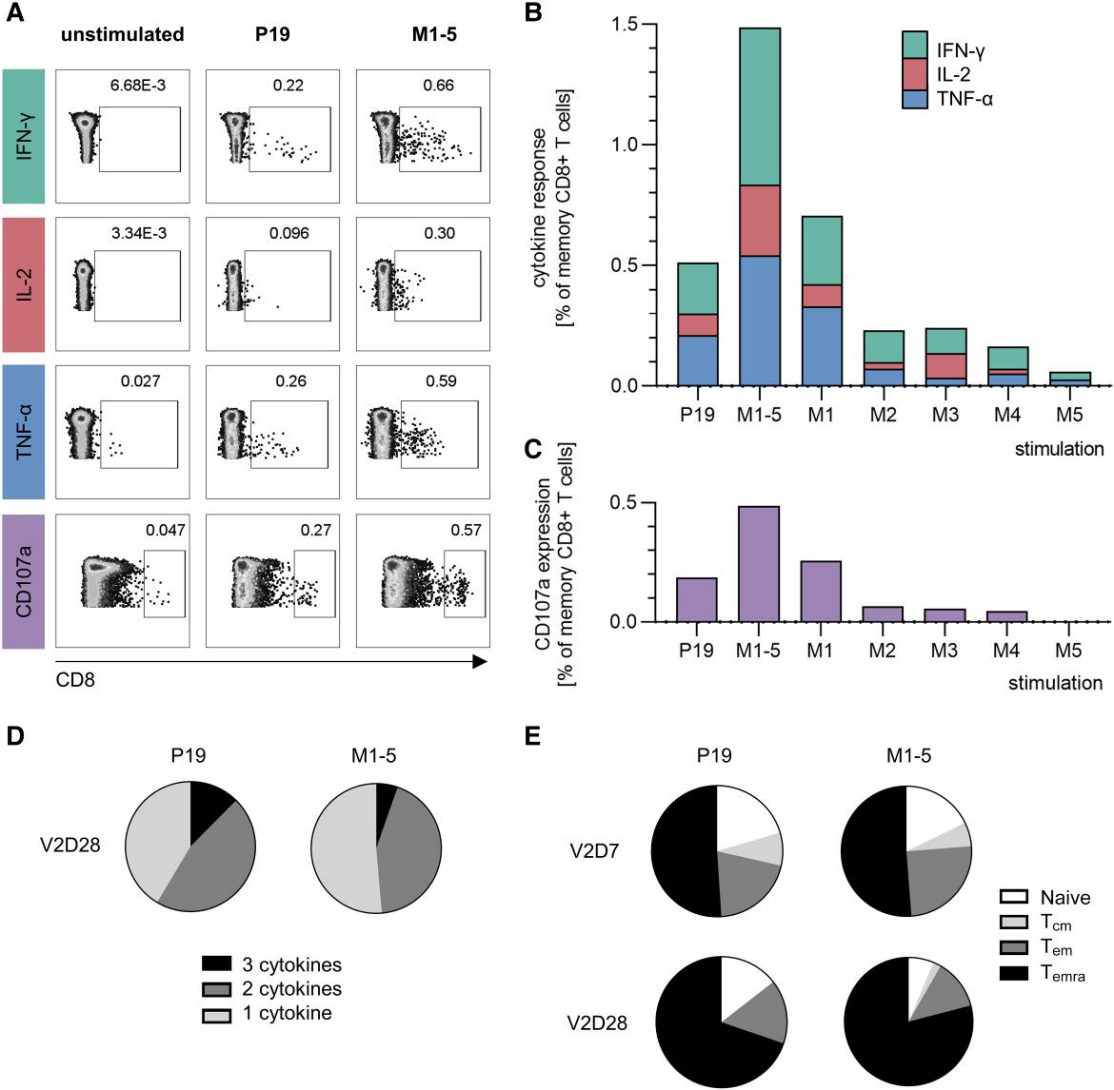
Functionality and memory phenotype of P19- compared to full MERS spike protein–specific CD8^+^ T cells. Interferon gamma (IFN-γ), interleukin 2 (IL-2), tumor necrosis factor alpha (TNF-α), and CD107a expression was measured using intracellular cytokine staining, after restimulation with the P19 peptide, M1–M5 pools separately and a combination of all pools (M1-5) covering the whole spike. *A*, Gatings are shown for the unstimulated negative control, after P19 and M1-5 restimulation at V2D28. Cytokine (*B*) and CD107a (*C*) positive cell frequencies are shown as percentages of total memory CD8^+^ T cells. *D*, Polyfunctionality of P19- and M1-5–specific CD8^+^ memory T cells was assessed using Boolean gating, showing those expressing 1 (IFN-γ^+^ or IL-2^+^ or TNF-α^+^), 2 (IFN-γ^+^IL-2^+^TNF-α^–^ or IFN-γ^+^IL-2^–^TNF-α^+^ or IFN-γ^−^IL-2^+^TNF-α^+^), or 3 cytokines (IFN-γ^+^IL-2^+^TNF-α^+^). *E*, Proportion of memory subsets out of total cytokine-positive cells at V2D7 and V2D28 (naive: CD45RO^–^CCR7^+^; central memory [T_cm_]: CD45RO^+^CCR7^+^; effector memory [T_em_]: CD45RO^+^CCR7^−^ ; effector memory reexpressing CD45RA [T_emra_]: CD45RO^−^CCR7^−^).

To further dissect the cytokine profile and CD4^+^/CD8^+^ bias of the T-cell response elicited by P19, we performed an ICS. After restimulation, we observed IFN-γ, IL-2, and TNF-α expression in memory CD8^+^ T cells (gating shown in Figure 2*A*), but not in memory CD4^+^ T cells (Supplementary Figure 2). Restimulation with individual M1–M5 OLP pools confirmed that the response was dominated by M1-specific T cells (V2D7 = 0.59%; V2D28 = 0.71%) (Figure 2*B*, Supplementary Figure 3). The frequency of total cytokine-secreting CD8^+^ T cells after restimulation with the single P19 peptide was comparable in magnitude (V2D7 = 0.48%; V2D28 = 0.51%). CD107a expression followed a similar pattern. At V2D28, the highest frequency was observed after M1-5 restimulation (0.49%), followed by M1 (0.26%) and P19 (0.19%), respectively (Figure 2*C*). The polyfunctional profile of P19-specific CD8^+^ T cells was similar to that of M1-5–specific CD8^+^ T cells (Figure 2*D*). We observed that the majority of P19-specific CD8^+^ T cells were memory cells (V2D7 = 80%; V2D28 = 86% of all P19-specific CD8^+^ T cells), resembling the findings of the M1-5–specific response (V2D7 = 82%; V2D28 = 94% of all M1-5–specific CD8^+^ T cells). The memory phenotype of P19-specific T cells shifted to a higher frequency of T effector memory cells reexpressing CD45RA (T_emra_) by V2D28 (75% of all P19-specific CD8^+^ T cells) compared to V2D7 (50%) (Figure 2*E*). A similar pattern was observed for M1-5–specific T cells, with a higher frequency of T_emras_ at V2D28 (79%) compared to V2D7 (52%).

Multiple sequence alignment revealed that P19 is specific to the MERS-S and not conserved in human betacoronaviruses (Supplementary Table 3). Next, we performed in silico epitope prediction of the complete MERS-S sequence for HLA-A*03:01 and HLA-B*35:01, the HLA-class I alleles for which the vaccinee was homozygous. Notably, an 11-mer (FPYQGDHGDMY) and a 13-mer epitope (FPYQGDHGDMYVY) within the P19 peptide were among the top 5 predicted HLA-B*35:01 MERS-S epitopes with the highest affinity (percentile rank = 0.01; Supplementary Table 4). One 9-mer epitope was identified in the HLA-A*03:01 prediction analysis, but with a lower affinity (percentile rank = 0.38; Supplementary Table 5).

To investigate if other vaccinees mounted a P19-specific response, we HLA-typed further 20 vaccinees of the study. By IFN-γ ELISpot we could show that the only other HLA-B*35:01 positive vaccinee of the clinical trial also responded to P19 (Supplementary Figure 4). Notably, P19 was not recognized by the 2 vaccinees who carried the HLA-A*03:01 but not the HLA-B*35:01 allele.

## DISCUSSION

The induction of functional and long-lasting immune responses is fundamental for successful vaccine design. The protective and durable nature of CD8^+^ T cells in MERS survivor studies makes them interesting targets for monitoring vaccine immunogenicity. Using an epitope-mapping approach, we identified a novel MERS-S–specific CD8^+^ T-cell epitope candidate (P19) in an individual vaccinated with MVA-MERS-S and could validate this finding in one further HLA-A*03:01/HLA-B*35:01-positive vaccinee. We observed a higher P19-specific T-cell response compared to all other peptides of the M1 pool and the M2–M5 pools, suggesting the potential immunodominance of this peptide within MERS-S.

P19-specific T cells were predominantly polyfunctional CD8^+^ T cells, and expressed CD107a, a marker of cytotoxicity. Polyfunctional CD8^+^ T cells can exert multiple effector functions and are associated with protection from disease. Interestingly, most P19-specific cells were T_emras_, with an increasing frequency from V2D14 to V2D28. CD8^+^ T_emras_ have been associated with protection against dengue and symptomatic H1N1 influenza and shown to be long lasting following dengue vaccination [10–11]. While the contribution of MERS-specific T_emras_ to protection remains elusive, previous studies have shown that memory T-cell responses, including IFN-γ–secreting T_emras,_ were detectable up to 6.9 years after infection [5, 7], suggesting that they may drive the maintenance of immune memory.

A beneficial characteristic of P19 for immunogenicity monitoring after MERS vaccination is its location in the N-terminal domain of the S1 subunit, outside the RBD, a region less prone to mutations or cross-reactivity. While the RBD is known to be highly immunogenic, mutations in the RBD have been implicated in the emergence of immune evasion in variants of SARS-CoV-2 [12]. These mutations may not only evade the humoral immune response but may also impact potential T-cell epitopes. Additionally, a report of the ChAdOx1-MERS vaccine clinical trial suggests that the RBD may have a role in T-cell cross-reactivity, given that preexisting T-cell responses detectable in a small number of vaccines were predominately directed against the RBD [13]. A phylogenetic analysis of 484 MERS-CoV isolates from humans and camels revealed no mutations in the P19 sequence [14]. Furthermore, our sequence alignment showed that P19 is not conserved among HCoVs. Taken together, the detected T-cell epitope mapping to a less mutagenic location of the spike with a highly specific sequence for MERS-CoV seems favorable for the longitudinal assessment of anti-MERS-CoV vaccine responses. This is particularly important given that preexisting immune responses to endemic HCoVs and SARS-CoV-2 are now highly prevalent and may interfere with immune monitoring during upcoming MERS vaccine trials.

In silico prediction revealed that P19 likely encompasses an HLA-B*35:01-restricted epitope. Notably, the allele frequency for HLA-B*35:01 ranges from 1.4% to 13.5% worldwide. HLA-B*35:01 is present in Germany (5.8%) but also in Saudi Arabia (3.0%), where MERS-CoV is endemic [15]. However, further studies are needed to identify the optimal epitope sequence, as MHC-I binding favors amino acid lengths of 9–11. A limitation of this study is that analyses were only performed in a limited number of vaccinees. However, we could show that P19 is recognized by all HLA-B*35:01-positive vaccinees of our phase 1a clinical trial. In conclusion, the identified CD8^+^ T-cell epitope may facilitate the implementation of tetramers to provide novel insights into the role of CD8^+^ T cells in anti-MERS-CoV immunity, which may further accelerate MERS-specific immune monitoring and the development of more efficacious vaccine candidates.

## Supplementary Data

Supplementary materials are available at *The Journal of Infectious Diseases* online (http://jid.oxfordjournals.org/). Supplementary materials consist of data provided by the author that are published to benefit the reader. The posted materials are not copyedited. The contents of all supplementary data are the sole responsibility of the authors. Questions or messages regarding errors should be addressed to the author.

## Supplementary Material

jiad612_Supplementary_Data

## Notes

***Acknowledgments.*** We thank the MVA-MERS-S study group members for their ongoing support and dedication. The following investigators are part of the MVA-MERS-S study group: Etienne Bartels, Monika Friedrich, Leonie M. Weskamm, Swantje Grundlach, and Joseph H. Poetsch (Institute for Infection Research and Vaccine Development, University Medical Centre Hamburg-Eppendorf, Hamburg, Germany; Department for Clinical Immunology of Infectious Diseases, Bernhard Nocht Institute for Tropical Medicine, Hamburg, Germany; German Center for Infection Research, partner site Hamburg-Lübeck-Borstel-Riems, Hamburg, Germany); Till Koch and Stefan Schmiedel (I. Department of Medicine, University Medical Center Hamburg-Eppendorf, Hamburg, Germany); Bart Haagmanns (Department of Viroscience, Erasmus Medical Centre, Rotterdam, The Netherlands); Thomas Hesterkamp (German Centre for Infection Research, Translational Project Management Office, Brunswick, Germany); Verena Krähling (Institute for Virology, Philipps University Marburg, Marburg, Germany; German Centre for Infection Research, partner site Gießen-Marburg-Langen, Marburg, Germany); and Asisa Volz (Institute of Virology, University of Veterinary Medicine Hannover, Foundation, Hanover, Germany; German Centre for Infection Research, partner site Hannover-Brunswick, Hanover, Germany). We thank all volunteers for their participation and their commitment and dedication to research against emerging coronaviruses. We thank Dr Vanessa A. Ditt und Melanie Kessler (Institute of Transfusion Medicine, University Medical Center Hamburg-Eppendorf) for the HLA typing of the vaccinees.

***Author contributions.*** Conceptualization: M. M. A., C. D., G. S., and S. B. Methodology: C. E. H. and L. M. Investigation: C. E. H., L. M., S. L., M. L. L., and the MVA-MERS-S Study Group. Formal analysis and visualization: C. E. H. and L. M. Writing–original draft: C. E. H. and L. M. Writing–review and editing: C. E. H., L. M., A. F., and M. M. A. Supervision: C. D., M. M. A., M. E. Z., and T. W.

***In memoriam.*** We dedicate this manuscript to Prof. Gerd Sutter who passed away on 31 October 2023, and thank him for his great contribution. He was instrumental in the design, generation, and preclinical evaluation of the MVA-based viral vector vaccines, as well as the conceptualization of the phase 1 clinical trials presented in this manuscript.

***Financial support*.** This work was funded by the German Center for Infection Research (DZIF Thematic Translational Unit [TTU] 01.908, TTU 01.702, and TTU 01.712). C. E. H. received a DZIF MD stipend (TI 07.003_Harrer).
