# Supplementary material for: Identification of a Spike-Specific CD8+ T-Cell Epitope Following Vaccination Against the Middle East Respiratory Syndrome Coronavirus in Humans
Source: J Infect Dis. 2024 Jan 9;230(2):e327–32. doi: 10.1093/infdis/jiad612 (PMC11326828; doi:10.1093/infdis/jiad612)
Supplement: jiad612_Supplementary_Data [file jiad612_supplementary_data.zip › Harrer_Supplementary_FigureLegends_Version2.docx]

**Supplementary Figure Legends**

Supplementary Figure 1: Intracellular staining gating strategy. Gating strategy for intracellular cytokine staining T cell assay (related to Fig. 2). Memory T cells were identified by exclusion CD45RO-CCR7+ naïve T cells.

Supplementary Figure 2: **Functionality of P19- compared to full MERS-spike-specific CD4+ T cells.** IFN-y, IL-2, TNF-a, and CD107a expression was measured using an intracellular cytokine staining, after re-stimulation with the P19 peptide, M1-M5 pools separately and a combination of all pools (M1-5) covering the whole spike (related to Fig. 2). Cytokine positive cell frequencies are shown as percentages of total memory CD4+ T cells.

**Supplementary Figure 3: Functionality of P19- compared to full MERS-spike-specific CD8+ T cells at V2D7.** IFN-γ, IL-2, TNF-α, and CD107a expression was measured using an intracellular cytokine staining, after re-stimulation with the P19 peptide, M1-M5 pools separately and a combination of all pools (M1-5) covering the whole spike. Cytokine **(A)** and CD107a **(B)** positive cell frequencies are shown as percentages of total memory CD8^+^ T cells.

**Supplementary Figure 4: IFN-γ ELISpot of HLA-B*35:01 and HLA-A*03:01 positive MVA-MERS-S phase 1a vaccinees.** Pictures below show representative wells and note the HLA-type of each vaccinee **(A)**. The bar graph shows the frequencies of T cells specific for the P19-peptide (black) and peptide pools M1-M5 (grey) covering the complete MERS-CoV spike protein of vaccine 4a who responded to P19 **(B)**.
