# Supplementary material for: Identification of a Spike-Specific CD8+ T-Cell Epitope Following Vaccination Against the Middle East Respiratory Syndrome Coronavirus in Humans
Source: J Infect Dis. 2024 Jan 9;230(2):e327–32. doi: 10.1093/infdis/jiad612 (PMC11326828; doi:10.1093/infdis/jiad612)
Supplement: jiad612_Supplementary_Data [file jiad612_supplementary_data.zip › Harrer_Supplementary_Table_1.docx]

**Supplementary Table 1:** List of customized MERS-S M1 single pool peptides P1-P65 assessed in this study. All MERS-S 15-mer peptides were customized by JPT and had a purity of > 70%.

| MERS-S Pool 1 Single Peptide | Sequence |
| --- | --- |
| MERS-S Pool 1, Peptide 1 | MIHSVFLLMFLLTPT |
| MERS-S Pool 1, Peptide 2 | VFLLMFLLTPTESYV |
| MERS-S Pool 1 Peptide 3 | MFLLTPTESYVDVGP |
| MERS-S Pool 1 Peptide 4 | TPTESYVDVGPDSVK |
| MERS-S Pool 1 Peptide 5 | SYVDVGPDSVKSACI |
| MERS-S Pool 1, Peptide 6 | VGPDSVKSACIEVDI |
| MERS-S Pool 1, Peptide 7 | SVKSACIEVDIQQTF |
| MERS-S Pool 1, Peptide 8 | ACIEVDIQQTFFDKT |
| MERS-S Pool 1, Peptide 9 | VDIQQTFFDKTWPRP |
| MERS-S Pool 1, Peptide 10 | QTFFDKTWPRPIDVS |
| MERS-S Pool 1, Peptide 11 | DKTWPRPIDVSKADG |
| MERS-S Pool 1, Peptide 12 | PRPIDVSKADGIIYP |
| MERS-S Pool 1, Peptide 13 | DVSKADGIIYPQGRT |
| MERS-S Pool 1, Peptide 14 | ADGIIYPQGRTYSNI |
| MERS-S Pool 1, Peptide 15 | IYPQGRTYSNITITY |
| MERS-S Pool 1, Peptide 16 | GRTYSNITITYQGLF |
| MERS-S Pool 1, Peptide 17 | SNITITYQGLFPYQG |
| MERS-S Pool 1, Peptide 18 | ITYQGLFPYQGDHGD |
| MERS-S Pool 1, Peptide 19 | GLFPYQGDHGDMYVY |
| MERS-S Pool 1, Peptide 20 | YQGDHGDMYVYSAGH |
| MERS-S Pool 1, Peptide 21 | HGDMYVYSAGHATGT |
| MERS-S Pool 1, Peptide 22 | YVYSAGHATGTTPQK |
| MERS-S Pool 1, Peptide 23 | AGHATGTTPQKLFVA |
| MERS-S Pool 1, Peptide 24 | TGTTPQKLFVANYSQ |
| MERS-S Pool 1, Peptide 25 | PQKLFVANYSQDVKQ |
| MERS-S Pool 1, Peptide 26 | FVANYSQDVKQFANG |
| MERS-S Pool 1, Peptide 27 | YSQDVKQFANGFVVR |
| MERS-S Pool 1, Peptide 28 | VKQFANGFVVRIGAA |
| MERS-S Pool 1, Peptide 29 | ANGFVVRIGAAANST |
| MERS-S Pool 1, Peptide 30 | VVRIGAAANSTGTVI |
| MERS-S Pool 1, Peptide 31 | GAAANSTGTVIISPS |
| MERS-S Pool 1, Peptide 32 | NSTGTVIISPSTSAT |
| MERS-S Pool 1, Peptide 33 | TVIISPSTSATIRKI |
| MERS-S Pool 1, Peptide 34 | SPSTSATIRKIYPAF |
| MERS-S Pool 1, Peptide 35 | SATIRKIYPAFMLGS |
| MERS-S Pool 1, Peptide 36 | RKIYPAFMLGSSVGN |
| MERS-S Pool 1, Peptide 37 | PAFMLGSSVGNFSDG |
| MERS-S Pool 1, Peptide 38 | LGSSVGNFSDGKMGR |
| MERS-S Pool 1, Peptide 39 | VGNFSDGKMGRFFNH |
| MERS-S Pool 1, Peptide 40 | SDGKMGRFFNHTLVL |
| MERS-S Pool 1, Peptide 41 | MGRFFNHTLVLLPDG |
| MERS-S Pool 1, Peptide 42 | FNHTLVLLPDGCGTL |
| MERS-S Pool 1, Peptide 43 | LVLLPDGCGTLLRAF |
| MERS-S Pool 1, Peptide 44 | PDGCGTLLRAFYCIL |
| MERS-S Pool 1, Peptide 45 | GTLLRAFYCILEPRS |
| MERS-S Pool 1, Peptide 46 | RAFYCILEPRSGNHC |
| MERS-S Pool 1, Peptide 47 | CILEPRSGNHCPAGN |
| MERS-S Pool 1, Peptide 48 | PRSGNHCPAGNSYTS |
| MERS-S Pool 1, Peptide 49 | NHCPAGNSYTSFATY |
| MERS-S Pool 1, Peptide 50 | AGNSYTSFATYHTPA |
| MERS-S Pool 1, Peptide 51 | YTSFATYHTPATDCS |
| MERS-S Pool 1, Peptide 52 | ATYHTPATDCSDGNY |
| MERS-S Pool 1, Peptide 53 | TPATDCSDGNYNRNA |
| MERS-S Pool 1, Peptide 54 | DCSDGNYNRNASLNS |
| MERS-S Pool 1, Peptide 55 | GNYNRNASLNSFKEY |
| MERS-S Pool 1, Peptide 56 | RNASLNSFKEYFNLR |
| MERS-S Pool 1, Peptide 57 | LNSFKEYFNLRNCTF |
| MERS-S Pool 1, Peptide 58 | KEYFNLRNCTFMYTY |
| MERS-S Pool 1, Peptide 59 | NLRNCTFMYTYNITE |
| MERS-S Pool 1, Peptide 60 | CTFMYTYNITEDEIL |
| MERS-S Pool 1, Peptide 61 | YTYNITEDEILEWFG |
| MERS-S Pool 1, Peptide 62 | ITEDEILEWFGITQT |
| MERS-S Pool 1, Peptide 63 | EILEWFGITQTAQGV |
| MERS-S Pool 1, Peptide 64 | WFGITQTAQGVHLFS |
| MERS-S Pool 1, Peptide 65 | TQTAQGVHLFSSRYV |
