# Supplementary material for: Identification of a Spike-Specific CD8+ T-Cell Epitope Following Vaccination Against the Middle East Respiratory Syndrome Coronavirus in Humans
Source: J Infect Dis. 2024 Jan 9;230(2):e327–32. doi: 10.1093/infdis/jiad612 (PMC11326828; doi:10.1093/infdis/jiad612)
Supplement: jiad612_Supplementary_Data [file jiad612_supplementary_data.zip › Harrer_Supplementary_Table_2.docx]

**Supplementary Table 2:** Characteristics of the 23 analysed participants enrolled in the MVA-MERS-S-study at baseline. The data in the right column depicts the characteristics of the study participant described in this report. Modified from Koch et al. (2020).

| **Characteristics** | **Total number** | **Percentage** | **Study participant** |
| --- | --- | --- | --- |
| **Age** |  |  |  |
| Mean (range) [yr] | 28.2 (18-47) | - | 18 |
| Median (SD) [yr] | 28 (7.5) | - |  |
| **Sex** |  |  |  |
| Female | 18 | 78.3% | Female |
| Male | 5 | 21.7% |  |
| **Body Mass Index** |  |  |  |
| Mean ± SD [kg/m2] | 23.4 ± 3.0 | - | 19 |
| **Ethnicity** |  |  |  |
| White | 22 | 95.7% | White |
| Black or African **American** | 0 | 0% |  |
| Native Hawaiian or **Pacific Islander** | 1 | 4.3% |  |
| Asian | 0 | 0% |  |
|  |  |  |  |
