# Supplementary material for: Identification of a Spike-Specific CD8+ T-Cell Epitope Following Vaccination Against the Middle East Respiratory Syndrome Coronavirus in Humans
Source: J Infect Dis. 2024 Jan 9;230(2):e327–32. doi: 10.1093/infdis/jiad612 (PMC11326828; doi:10.1093/infdis/jiad612)
Supplement: jiad612_Supplementary_Data [file jiad612_supplementary_data.zip › Harrer_Supplementary_Table_3.docx]

**Supplementary Table 3:** MERS-S epitopes predicted to be strong HLA-B*35:01 binders (% rank < 0.5) using the NetMHCpan - 4.1 software. Epitopes overlapping with P19 are highlighted in grey.

| **allele** | **start** | **end** | **length** | **peptide** | **score** | **percentile rank** |
| --- | --- | --- | --- | --- | --- | --- |
| HLA-B*35:01 | 284 | 292 | 9 | LPVYDTIKY | 0.992963 | 0.01 |
| HLA-B*35:01 | 75 | 85 | 11 | FPYQGDHGDMY | 0.988827 | 0.01 |
| HLA-B*35:01 | 970 | 978 | 9 | IPFAQSIFY | 0.988076 | 0.01 |
| HLA-B*35:01 | 786 | 794 | 9 | FSFGVTQEY | 0.978991 | 0.01 |
| HLA-B*35:01 | 75 | 87 | 13 | FPYQGDHGDMYVY | 0.975461 | 0.01 |
| HLA-B*35:01 | 633 | 641 | 9 | DAYQNLVGY | 0.936625 | 0.03 |
| HLA-B*35:01 | 1036 | 1044 | 9 | LASELSNTF | 0.9327 | 0.03 |
| HLA-B*35:01 | 1142 | 1153 | 12 | YPSNHIEVVSAY | 0.931641 | 0.03 |
| HLA-B*35:01 | 766 | 777 | 12 | HPIQVDQLNSSY | 0.911709 | 0.04 |
| HLA-B*35:01 | 569 | 577 | 9 | MGFGITVQY | 0.876102 | 0.05 |
| HLA-B*35:01 | 284 | 293 | 10 | LPVYDTIKYY | 0.872338 | 0.05 |
| HLA-B*35:01 | 555 | 563 | 9 | VASGSTVAM | 0.851512 | 0.06 |
| HLA-B*35:01 | 75 | 84 | 10 | FPYQGDHGDM | 0.828621 | 0.07 |
| HLA-B*35:01 | 1195 | 1204 | 10 | EPITSLNTKY | 0.77512 | 0.09 |
| HLA-B*35:01 | 655 | 663 | 9 | VSVPVSVIY | 0.748666 | 0.1 |
| HLA-B*35:01 | 1142 | 1150 | 9 | YPSNHIEVV | 0.740425 | 0.1 |
| HLA-B*35:01 | 10 | 18 | 9 | FLLTPTESY | 0.729393 | 0.11 |
| HLA-B*35:01 | 199 | 207 | 9 | NSYTSFATY | 0.729038 | 0.11 |
| HLA-B*35:01 | 782 | 794 | 13 | IPTNFSFGVTQEY | 0.715325 | 0.12 |
| HLA-B*35:01 | 58 | 71 | 14 | YPQGRTYSNITITY | 0.695981 | 0.13 |
| HLA-B*35:01 | 283 | 292 | 10 | TLPVYDTIKY | 0.67593 | 0.13 |
| HLA-B*35:01 | 429 | 438 | 10 | SPAAIASNCY | 0.673934 | 0.13 |
| HLA-B*35:01 | 56 | 64 | 9 | IIYPQGRTY | 0.669525 | 0.13 |
| HLA-B*35:01 | 1204 | 1211 | 8 | YVAPQVTY | 0.654079 | 0.13 |
| HLA-B*35:01 | 681 | 689 | 9 | HISSTMSQY | 0.637099 | 0.15 |
| HLA-B*35:01 | 769 | 777 | 9 | QVDQLNSSY | 0.629193 | 0.15 |
| HLA-B*35:01 | 533 | 541 | 9 | TVWEDGDYY | 0.625745 | 0.15 |
| HLA-B*35:01 | 514 | 523 | 10 | VPQLVNANQY | 0.618331 | 0.15 |
| HLA-B*35:01 | 281 | 292 | 12 | FATLPVYDTIKY | 0.614785 | 0.16 |
| HLA-B*35:01 | 780 | 788 | 9 | LSIPTNFSF | 0.613408 | 0.16 |
| HLA-B*35:01 | 967 | 978 | 12 | FAAIPFAQSIFY | 0.59598 | 0.17 |
| HLA-B*35:01 | 195 | 204 | 10 | CPAGNSYTSF | 0.5834 | 0.17 |
| HLA-B*35:01 | 561 | 569 | 9 | VAMTEQLQM | 0.565008 | 0.19 |
| HLA-B*35:01 | 1256 | 1264 | 9 | NTTLLDLTY | 0.561576 | 0.19 |
| HLA-B*35:01 | 96 | 105 | 10 | TPQKLFVANY | 0.540863 | 0.21 |
| HLA-B*35:01 | 1184 | 1192 | 9 | WSYTGSSFY | 0.535099 | 0.22 |
| HLA-B*35:01 | 279 | 287 | 9 | FQFATLPVY | 0.531689 | 0.22 |
| HLA-B*35:01 | 64 | 71 | 8 | YSNITITY | 0.51915 | 0.22 |
| HLA-B*35:01 | 386 | 397 | 12 | SPLLSGTPPQVY | 0.509222 | 0.23 |
| HLA-B*35:01 | 969 | 978 | 10 | AIPFAQSIFY | 0.502182 | 0.23 |
| HLA-B*35:01 | 1192 | 1200 | 9 | YAPEPITSL | 0.499505 | 0.23 |
| HLA-B*35:01 | 308 | 316 | 9 | KAWAAFYVY | 0.497469 | 0.23 |
| HLA-B*35:01 | 319 | 327 | 9 | QPLTFLLDF | 0.496951 | 0.23 |
| HLA-B*35:01 | 448 | 456 | 9 | YPLSMKSDL | 0.456832 | 0.26 |
| HLA-B*35:01 | 46 | 58 | 13 | RPIDVSKADGIIY | 0.456191 | 0.26 |
| HLA-B*35:01 | 1272 | 1280 | 9 | VVKALNESY | 0.453888 | 0.27 |
| HLA-B*35:01 | 968 | 978 | 11 | AAIPFAQSIFY | 0.44856 | 0.27 |
| HLA-B*35:01 | 1145 | 1153 | 9 | NHIEVVSAY | 0.440703 | 0.28 |
| HLA-B*35:01 | 343 | 351 | 9 | DLSQLHCSY | 0.432436 | 0.29 |
| HLA-B*35:01 | 766 | 773 | 8 | HPIQVDQL | 0.423642 | 0.3 |
| HLA-B*35:01 | 900 | 909 | 10 | IADPGYMQGY | 0.410211 | 0.31 |
| HLA-B*35:01 | 530 | 541 | 12 | VPSTVWEDGDYY | 0.409431 | 0.31 |
| HLA-B*35:01 | 785 | 794 | 10 | NFSFGVTQEY | 0.404677 | 0.31 |
| HLA-B*35:01 | 633 | 642 | 10 | DAYQNLVGYY | 0.403071 | 0.32 |
| HLA-B*35:01 | 352 | 361 | 10 | ESFDVESGVY | 0.400968 | 0.32 |
| HLA-B*35:01 | 172 | 180 | 9 | LPDGCGTLL | 0.400751 | 0.32 |
| HLA-B*35:01 | 970 | 977 | 8 | IPFAQSIF | 0.389297 | 0.33 |
| HLA-B*35:01 | 69 | 77 | 9 | ITYQGLFPY | 0.378013 | 0.34 |
| HLA-B*35:01 | 1163 | 1171 | 9 | NCIAPVNGY | 0.372321 | 0.35 |
| HLA-B*35:01 | 632 | 641 | 10 | YDAYQNLVGY | 0.369111 | 0.35 |
| HLA-B*35:01 | 459 | 467 | 9 | SSAGPISQF | 0.358234 | 0.36 |
| HLA-B*35:01 | 63 | 71 | 9 | TYSNITITY | 0.351844 | 0.37 |
| HLA-B*35:01 | 489 | 497 | 9 | TTITKPLKY | 0.335614 | 0.39 |
| HLA-B*35:01 | 766 | 778 | 13 | HPIQVDQLNSSYF | 0.331209 | 0.39 |
| HLA-B*35:01 | 738 | 745 | 8 | LPDTPSTL | 0.319647 | 0.4 |
| HLA-B*35:01 | 729 | 738 | 10 | LPLGQSLCAL | 0.308649 | 0.41 |
| HLA-B*35:01 | 389 | 397 | 9 | LSGTPPQVY | 0.307979 | 0.41 |
| HLA-B*35:01 | 282 | 292 | 11 | ATLPVYDTIKY | 0.30616 | 0.41 |
| HLA-B*35:01 | 935 | 943 | 9 | LPPLMDVNM | 0.30267 | 0.41 |
| HLA-B*35:01 | 222 | 231 | 10 | NASLNSFKEY | 0.299597 | 0.42 |
| HLA-B*35:01 | 74 | 85 | 12 | LFPYQGDHGDMY | 0.297851 | 0.42 |
| HLA-B*35:01 | 258 | 266 | 9 | QTAQGVHLF | 0.297206 | 0.42 |
| HLA-B*35:01 | 491 | 499 | 9 | ITKPLKYSY | 0.291933 | 0.43 |
| HLA-B*35:01 | 1193 | 1204 | 12 | APEPITSLNTKY | 0.291805 | 0.43 |
| HLA-B*35:01 | 75 | 86 | 12 | FPYQGDHGDMYV | 0.269157 | 0.46 |
| HLA-B*35:01 | 709 | 717 | 9 | TPVGCVLGL | 0.265006 | 0.48 |
