# Supplementary material for: Identification of a Spike-Specific CD8+ T-Cell Epitope Following Vaccination Against the Middle East Respiratory Syndrome Coronavirus in Humans
Source: J Infect Dis. 2024 Jan 9;230(2):e327–32. doi: 10.1093/infdis/jiad612 (PMC11326828; doi:10.1093/infdis/jiad612)
Supplement: jiad612_Supplementary_Data [file jiad612_supplementary_data.zip › Harrer_Supplementary_Table_4.docx]

**Supplementary Table 4:** MERS-S epitopes predicted to be strong HLA-A*03:01 binders (% rank < 0.5) using the NetMHCpan - 4.1 software. Epitopes overlapping with P19 are highlighted in grey.

| **allele** | **start** | **end** | **length** | **peptide** | **score** | **Percentile rank** |
| --- | --- | --- | --- | --- | --- | --- |
| HLA-A*03:01 | 42 | 52 | 11 | KTWPRPIDVSK | 0.919727 | 0.02 |
| HLA-A*03:01 | 62 | 71 | 10 | RTYSNITITY | 0.838926 | 0.05 |
| HLA-A*03:01 | 359 | 369 | 11 | GVYSVSSFEAK | 0.827648 | 0.06 |
| HLA-A*03:01 | 1275 | 1284 | 10 | ALNESYIDLK | 0.804656 | 0.08 |
| HLA-A*03:01 | 758 | 766 | 9 | RLASIAFNH | 0.72362 | 0.14 |
| HLA-A*03:01 | 1025 | 1035 | 11 | AVNNNAQALSK | 0.715454 | 0.15 |
| HLA-A*03:01 | 1102 | 1112 | 11 | KVNECVKAQSK | 0.691151 | 0.17 |
| HLA-A*03:01 | 482 | 493 | 12 | ATVPHNLTTITK | 0.68517 | 0.17 |
| HLA-A*03:01 | 56 | 64 | 9 | IIYPQGRTY | 0.674618 | 0.18 |
| HLA-A*03:01 | 282 | 291 | 10 | ATLPVYDTIK | 0.640538 | 0.21 |
| HLA-A*03:01 | 459 | 470 | 12 | SSAGPISQFNYK | 0.622 | 0.23 |
| HLA-A*03:01 | 1092 | 1100 | 9 | AALSAQLAK | 0.621145 | 0.23 |
| HLA-A*03:01 | 493 | 502 | 10 | KPLKYSYINK | 0.538059 | 0.29 |
| HLA-A*03:01 | 299 | 308 | 10 | SIRSIQSDRK | 0.515772 | 0.32 |
| HLA-A*03:01 | 687 | 698 | 12 | SQYSRSTRSMLK | 0.504269 | 0.33 |
| HLA-A*03:01 | 1093 | 1102 | 10 | ALSAQLAKDK | 0.489793 | 0.35 |
| HLA-A*03:01 | 1266 | 1274 | 9 | MLSLQQVVK | 0.481924 | 0.37 |
| HLA-A*03:01 | 69 | 77 | 9 | ITYQGLFPY | 0.471845 | 0.38 |
| HLA-A*03:01 | 489 | 497 | 9 | TTITKPLKY | 0.456958 | 0.4 |
| HLA-A*03:01 | 845 | 854 | 10 | SVRNLFASVK | 0.45648 | 0.4 |
| HLA-A*03:01 | 807 | 816 | 10 | KQYVCNGFQK | 0.453291 | 0.4 |
| HLA-A*03:01 | 308 | 317 | 10 | KAWAAFYVYK | 0.407662 | 0.47 |
| HLA-A*03:01 | 130 | 142 | 13 | VIISPSTSATIRK | 0.406598 | 0.47 |
