# Supplementary material for: Identification of a Spike-Specific CD8+ T-Cell Epitope Following Vaccination Against the Middle East Respiratory Syndrome Coronavirus in Humans
Source: J Infect Dis. 2024 Jan 9;230(2):e327–32. doi: 10.1093/infdis/jiad612 (PMC11326828; doi:10.1093/infdis/jiad612)
Supplement: jiad612_Supplementary_Data [file jiad612_supplementary_data.zip › Harrer_Supplementary_Table_5.docx]

**Supplementary Table 5:** Multiple Sequence alignment of human *Betacoronavirus* spike proteins using the Clustal Omega we server. The MERS-S P19 peptide is highlighted in yellow.

hCoV-OC43 ----MFLILLISLPT-AFAVIGDLKCTSDNIN----D--KDTGPPPISTDTVDVTNGLGT 49

hCoV-HKU1 -----MLLIIFILPT-TLAVIGDFNCTNFAIN----D--KNTTVPRISEYVVDVSYGLGT 48

MERS-CoV MIHSVFLLMFLLTPTESYVDVGPDSVKSACIEVDIQQTFFDKTW---PRP-IDVSKADGI 56

SARS-CoV-2 ----MFVFLVLLPLV-S----------SQCVN--LTT--RTQLP---PAY--TNSFTRGV 36

SARS-CoV ----MFIFLLFLTLT-S----------GSDLD--RCTTFDDVQA---PNYTQHTSSMRGV 40

::::.: . : . :: : *

hCoV-OC43 YYVLDRVYLNTTLFLNGYYPTSGSTYRNM------ALKGSVLLSR--LWFKPPFLSDFIN 101

hCoV-HKU1 YYILDRVYLNTTILFTGYFPKSGANFRDL------SLKGTTYLST--LWYQKPFLSDFNN 100

MERS-CoV IYPQGRTYSNITITYQGLF-PYQGDHGDMYVYSAGHATGTTPQKLFVANYSQD-VKQFAN 114

SARS-CoV-2 YYPDKVFRSSVLHSTQDLFLPFFSNVT---WFHAIHVSGTNGTKR----FDNP-VLPFND 88

SARS-CoV YYPDEIFRSDTLYLTQDLFLPFYSNVT---GFHTIN-------HT----FDNP-VIPFKD 85

* . . : . :. : * :

hCoV-OC43 GIFAKVKNTKVIKDRVM---------YSEFPAITIGSTFVNTSY---------SVVVQPR 143

hCoV-HKU1 GIFSRVKNTKLYVNKTL---------YSEFSTIVIGSVFINNSY---------TIVVQPH 142

MERS-CoV GFVVRIGAAANSTGTVIISPSTSATIRKIYPAFMLGSSVGNFSDGKMGRFFNHTLVLLPD 174

SARS-CoV-2 GVYFASTE-----------------KSNIIRGWIFGTTLDSKTQ---------SLLIVNN 122

SARS-CoV GIYFAATE-----------------KSNVVRGWVFGSTMNNKSQ---------SVIIINN 119

*. . :*: . . : ::::

hCoV-OC43 TINSTQDGDNKLQGLLEVSVCQYNMCEYPQTICHPNLGNHRKELWHLDTGVVSC------ 197

hCoV-HKU1 ------------NGVLEITACQYTMCEYPHTICKS-KGSSRNESWHFDKSEPLC------ 183

MERS-CoV -------G-----CGTLLRAF--YCILEPRSGNHCPAGNSYTSFATYHTPATDCSDGNYN 220

SARS-CoV-2 -------A-----TNVVIKVCEFQFCNDPFLGVYYHKNN--------------------- 149

SARS-CoV -------S-----TNVVIRACNFELCDNPFFAVSKPMGT--------------------- 146

: . * ..

hCoV-OC43 -------------LYKRNFTYDVN--AD-----YLY------FHFYQEGGTFYAYFTDT- 230

hCoV-HKU1 -------------LFKKNFTYNVS--TD-----FLY------FHFYQERGTFYAYYADS- 216

MERS-CoV RNASLNSFKEYFNLRNCTFMYTYNITEDEILEWFGITQTAQG-VHLFSSRYVDLYGGN-- 277

SARS-CoV-2 KSWMESEFRVYSSANNCTFEYVSQPFLMDLEGKQGNFKNLREFVFKNIDGYFKIYSKHTP 209

SARS-CoV ----QTHTMIFDNAFNCTFEYISDAFSLDVSEKSGNFKHLREFVFKNKDGFLYVYKGYQP 202

: .* * . . . *

hCoV-OC43 ---------GVV-TKFLFNVYLGMALSHYYVMPLTCNS------------KLTLEYWVTP 268

hCoV-HKU1 ---------GMP-TTFLFSLYLGTLLSHYYVLPLTCNA------ISSNTDNETLQYWVTP 260

MERS-CoV -------------MFQFATLPVYDTIKYYSIIPHSIRS---IQSDRKAW----AAFYVYK 317

SARS-CoV-2 INLVRDLPQGFSALEPLVDLPIGINITRFQTLLALHRSYLTPGDSSSGWTAGAAAYYVGY 269

SARS-CoV IDVVRDLPSGFNTLKPIFKLPLGINITNFRAILTAF------SPAQDTWGTSAAAYFVGY 256

: : : :. : : ::*

hCoV-OC43 LTSRQYLLAFNQDGIIFNAVDCMSDFMSEIKCKTQSIAPPTGVYELNGYTVQPIADVYRR 328

hCoV-HKU1 LSKRQYLLKFDNRGVITNAVDCSSSFFSEIQCKTKSLLPNTGVYDLSGFTVKPVATVHRR 320

MERS-CoV LQPLTFLLDFSVDGYIRRAIDCGFNDLSQLHCSYESFDVESGVYSVSSFEAKPSGSVVEQ 377

SARS-CoV-2 LQPRTFLLKYNENGTITDAVDCALDPLSETKCTLKSFTVEKGIYQTSNFRVQPTESIVRF 329

SARS-CoV LKPTTFMLKYDENGTITDAVDCSQNPLAELKCSVKSFEIDKGIYQTSNFRVVPSGDVVRF 316

* ::* :. * * *:** . ::: :*. :*: .*:*. ..: . * : .

hCoV-OC43 KPNLPNCNIEAWLNDKSVPSPLNWERKTFSNCNFNMSSLMSFIQADSFTCNNIDAAKIYG 388

hCoV-HKU1 IPDLPDCDIDKWLNNFNVPSPLNWERKIFSNCNFNLSTLLRLVHTDSFSCNNFDESKIYG 380

MERS-CoV AEG-VECDFSPLLSG-TPPQVYNFKRLVFTNCNYNLTKLLSLFSVNDFTCSQISPAAIAS 435

SARS-CoV-2 PNITNLCPFGEVFNATRFASVYAWNRKRISNCVADYSVLYNSASFSTFKCYGVSPTKLND 389

SARS-CoV PNITNLCPFGEVFNATKFPSVYAWERKKISNCVADYSVLYNSTFFSTFKCYGVSATKLND 376

* : :. . ::* ::** : : * . *.* .. : : .

hCoV-OC43 MCFSSITIDKFAIPNGRKVDLQLGNLGYLQSFNYRIDTTATSCQLYYNLPAANVSVSRFN 448

hCoV-HKU1 SCFKSIVLDKFAIPNSRRSDLQLGSSGFLQSSNYKIDTTSSSCQLYYSLPAINVTINNYN 440

MERS-CoV NCYSSLILDYFSYPLSMKSDLSVSSAGPISQFNYKQSFSNPTCLILATVPHNLTTITKP- 494

SARS-CoV-2 LCFTNVYADSFVIRGDEVRQIAPGQTGKIADYNYKLPDDFTGCVIAWNSNNLDSKVGGN- 448

SARS-CoV LCFSNVYADSFVVKGDDVRQIAPGQTGVIADYNYKLPDDFMGCVLAWNTRNIDATSTGN- 435

*:..: * * . :: .. * : . **: * : . .

hCoV-OC43 PSTWNKRFGFIEDSVFKPRPAGVLTNHDVVYAQHCFKAPKNFCPCKLNGSCVGSGPGKNN 508

hCoV-HKU1 PSSWNRRYGFNNF---------NLSSHSVVYSRYCFSVNNTFCPCAKPSFAS-SCKSHKP 490

MERS-CoV -----LKYSYINKCSR------LLSDDRTEVPQLVNAN--QYSPCVSI----------V- 530

SARS-CoV-2 -----YNYLYRLFRKS------NLKPFERDISTEIYQA--GSTPCNGV----------E- 484

SARS-CoV -----YNYKYRYLRHG------KLRPFERDISNVPFSP--DGKPCTP-----------P- 470

.: : * **

hCoV-OC43 GIGTCPAGTNYLTCD------------NLCTPDPITFTGTYKCPQTKSLVGIGEHCSGLA 556

hCoV-HKU1 PSASCPIGTNYRSCESTTVLDHTDWCRCSCLPDPITAYDPRSCSQKKSLVGVGEHCAGFG 550

MERS-CoV -PST--------------VWEDGDYYRKQL--SPLEGGGWLVA-------------SGST 560

SARS-CoV-2 -GFNC-------------YF-------------PLQSYGFQPT-------------NGVG 504

SARS-CoV -ALNC-------------YW-------------PLNDYGFYTT-------------TGIG 490

. *: . *

hCoV-OC43 VKSDYCGG------NSCTCRPQAFLGWSADSCLQGDKCNIFANFILHDVNSGLTCST--D 608

hCoV-HKU1 VDEEKCGVLDGSYNVSCLCSTDAFLGWSYDTCVSNNRCNIFSNFILNGINSGTTCSN--D 608

MERS-CoV VAMT----------------EQLQMGF--------------GITVQYGTDTNSVCPKLEF 590

SARS-CoV-2 YQPY----------------RVVVLSF--------------ELL----HAPATVCGP--- 527

SARS-CoV YQPY----------------RVVVLSF--------------ELL----NAPATVCGP--- 513

:.: .*

hCoV-OC43 LQKANTDIILGVCVNYDLYGILGQGIFVEVNATYYNSWQNLLYDSNGNLY-GFRDYITNR 667

hCoV-HKU1 LLQPNTEVYTDVCVDYDLYGITGQGIFKEVSAVYYNSWQNLLYDSNGNII-GFKDFVTNK 667

MERS-CoV ANDTKIASQLGNCVEYSLYGVSGRGVFQNCTAVGV-RQQRFVYDAYQNLVGYYSD--DGN 647

SARS-CoV-2 --KKSTNLVKNKCVNFNFNGLTGTGVLTESNKKFL-PFQQFGRDIADTT-DAVRDPQTLE 583

SARS-CoV --KLSTDLIKNQCVNFNFNGLTGTGVLTPSSKRFQ-PFQQFGRDVSDFT-DSVRDPKTSE 569

. . . **::.: *: * *:: . *.: * * .

hCoV-OC43 TFMIRSCYSGRVSAAFH--ANSSEPALLFRNIKCNYVFNNSLTRQLQPI----------N 715

hCoV-HKU1 TYNIFPCYAGRVSAAFH--QNASSLALLYRNLKCSYVLNNIS---LATQ----------P 712

MERS-CoV YYCLRACVSVPVSVIYD--KETKTHATLFGSVACEHISSTMSQYSRSTRSMLKRRDSTYG 705

SARS-CoV-2 ILDITPCSFGGVSVITPGTNTSNQVAVLYQDVNCTEVPVAIHADQLT--PTWRVYSTGSN 641

SARS-CoV ILDISPCSFGGVSVITPGTNASSEVAVLYQDVNCTDVSTAIHADQLT--PAWRIYSTGNN 627

: * **. :. * *: .: * :

hCoV-OC43 YFDSYLGCVVNAYNSTAISVQTCDLTVGSGYCVDYS-----KNRRSRGAITTGYRFTNFE 770

hCoV-HKU1 YFDSYLGCVFNADNLTDYSVSSCALRMGSGFCVDYNSPSFSSSRRKRRSISASYRFVTFE 772

MERS-CoV PLQTPVGCVLGLVNSS-LFVEDCKLPLGQSLCALPDTPSTLTPRSVRSVPGE-MRLASI- 762

SARS-CoV-2 VFQTRAGCLIGAEHVN--NSYECDIPIGAGICASYQTQT-NSPRRARSVASQ-SI---I- 693

SARS-CoV VFQTQAGCLIGAEHVD--TSYECDIPIGAGICASYHTVS-L----LRSTSQK-SI---V- 675

::: **:.. : * : :* . *. * .

hCoV-OC43 PFTVNS-VNDSLEPVGGLYEIQIPSEFTIGNMVEFIQTSSPKVTIDCAAFVCGDYAACKS 829

hCoV-HKU1 PFNVSF-VNDSIESVGGLYEIKIPTNFTIVGQEEFIQTNSPKVTIDCSLFVCSNYAACHD 831

MERS-CoV AFNHPIQV-DQL--NSSYFKLSIPTNFSFGVTQEYIQTTIQKVTVDCKQYVCNGFQKCEQ 819

SARS-CoV-2 AYTMSLGAENSV--AYSNNSIAIPTNFTISVTTEILPVSMTKTSVDCTMYICGDSTECSN 751

SARS-CoV AYTMSLGADSSI--AYSNNTIAIPTNFSISITTEVMPVSMAKTSVDCNMYICGDSTECAN 733

:. . ..: . : **::*:: * : .. *.::** ::*.. * .

hCoV-OC43 QLVEYGSFCDNINAILTEVNELLDTTQLQVANSLMNGVTLSTKLKDGVNFNVDDINFSPV 889

hCoV-HKU1 LLSEYGTFCDNINSILDEVNGLLDTTQLHVADTLMQGVTLSSNLNTNLHFDVDNINFKSL 891

MERS-CoV LLREYGQFCSKINQALHGANLRQDDSVRNLFASVKSSQSSPIIPGFG-----GDFNLTLL 874

SARS-CoV-2 LLLQYGSFCTQLNRALTGIAVEQDKNTQEVFAQVKQIYKTPPIKDFG-----GF-NFSQI 805

SARS-CoV LLLQYGSFCTQLNRALSGIAAEQDRNTREVFAQVKQMYKTPTLKYFG-----GF-NFSQI 787

* :** ** ::* * * . .: : . . . . *:. :

hCoV-OC43 LGCLGSECSKASSRSAIEDLLFDKVKLSDVGFVEAYNNCT--GGAEIRDLICVQSYKGIK 947

hCoV-HKU1 VGCLGPHCGS-SSRSFFEDLLFDKVKLSDVGFVEAYNNCT--GGSEIRDLLCVQSFNGIK 948

MERS-CoV EP-VSISTGSRSARSAIEDLLFDKVTIADPGYMQGYDDCMQQGPASARDLICAQYVAGYK 933

SARS-CoV-2 LP-DP---SKPSKRSFIEDLLFNKVTLADAGFIKQYGDCL--GDIAARDLICAQKFNGLT 859

SARS-CoV LP-DP---LKPTKRSFIEDLLFNKVTLADAGFMKQYGECL--GDINARDLICAQKFNGLT 841

. : ** :*****:**.::* *::: *.:* * ***:*.* * .

hCoV-OC43 VLPPLLSENQISGYTLAATSASLFPPWTAA----AGVPFYLNVQYRINGLGVTMDVLSQN 1003

hCoV-HKU1 VLPPILSESQISGYTTAATVAAMFPPWSAA----AGIPFSLNVQYRINGLGVTMDVLNKN 1004

MERS-CoV VLPPLMDVNMEAAYTSSLLGSIAGVGWTAGLSSFAAIPFAQSIFYRLNGVGITQQVLSEN 993

SARS-CoV-2 VLPPLLTDEMIAQYTSALLAGTITSGWTFGAGAALQIPFAMQMAYRFNGIGVTQNVLYEN 919

SARS-CoV VLPPLLTDDMIAAYTAALVSGTATAGWTFGAGAALQIPFAMQMAYRFNGIGVTQNVLYEN 901

****:: . : ** : . *: . :** .: **:**:*:* :** :*

hCoV-OC43 QKLIANAFNNALYAIQEGFDATNSALVKIQAVVNANAEALNNLLQQLSNRFGAISASLQE 1063

hCoV-HKU1 QKLIATAFNNALLSIQNGFSATNSALAKIQSVVNSNAQALNSLLQQLFNKFGAISSSLQE 1064

MERS-CoV QKLIANKFNQALGAMQTGFTTTNEAFQKVQDAVNNNAQALSKLASELSNTFGAISASIGD 1053

SARS-CoV-2 QKLIANQFNSAIGKIQDSLSSTASALGKLQDVVNQNAQALNTLVKQLSSNFGAISSVLND 979

SARS-CoV QKQIANQFNKAISQIQESLTTTSTALGKLQDVVNQNAQALNTLVKQLSSNFGAISSVLND 961

** **. **.*: :* .: :* *: *:* .** **:**..* .:* . *****: : :

hCoV-OC43 ILSRLDALEAEAQIDRLINGRLTALNAYVSQQLSDSTLVKFSAAQAMEKVNECVKSQSSR 1123

hCoV-HKU1 ILSRLDALEAQVQIDRLINGRLTALNAYVSQQLSDISLVKFGAALAMEKVNECVKSQSPR 1124

MERS-CoV IIQRLDVLEQDAQIDRLINGRLTTLNAFVAQQLVRSESAALSAQLAKDKVNECVKAQSKR 1113

SARS-CoV-2 ILSRLDKVEAEVQIDRLITGRLQSLQTYVTQQLIRAAEIRASANLAATKMSECVLGQSKR 1039

SARS-CoV ILSRLDKVEAEVQIDRLITGRLQSLQTYVTQQLIRAAEIRASANLAA-TMSECVLGQSKR 1020

*:.*** :* :.******.*** :*:::*:*** .* * .:.*** .** *

hCoV-OC43 INFCGNGNHIISLVQNAPYGLYFIHFSYVPTKYVTARVSPGLCIAGDR--GIAPKSGYFV 1181

hCoV-HKU1 INFCGNGNHILSLVQNAPYGLLFMHFSYKPISFKTVLVSPGLCISGDV--GIAPKQGYFI 1182

MERS-CoV SGFCGQGTHIVSFVVNAPNGLYFMHVGYYPSNHIEVVSAYGLCDAANPTNCIAPVNGYFI 1173

SARS-CoV-2 VDFCGKGYHLMSFPQSAPHGVVFLHVTYVPAQEKNFTTAPAICHDGKA---HFPREGVFV 1096

SARS-CoV VDFCGKGYHLMSFPQAAPHGVVFLHVTYVPSQERNFTTAPAICHEGKA---YFPREGVFV 1077

.***:* *::*: ** *: *:*. * * . : .:* .. * .* *:

hCoV-OC43 NV-----NNTWMYTGSGYYYPEPITENNVVVMSTCAVNYTKAPYVM--LNTSIPNLPDFK 1234

hCoV-HKU1 KH-----NDHWMFTGSSYYYPEPISDKNVVFMNTCSVNFTKAPLVY--LNHSVPKLSDFE 1235

MERS-CoV KTNNTRIVDEWSYTGSSFYAPEPITSLNTKYVAPQVTYQN-ISTNLPPPLLGNSTGIDFQ 1232

SARS-CoV-2 SN-----GTHWFVTQRNFYEPQIITTDNTFVSGNCDVVIGIVNNTVYDPLQ--PELDSFK 1149

SARS-CoV FN-----GTSWFITQRNFFSPQIITTDNTFVSGNCDVVIGIINNTVYDPLQ--PELDSFK 1130

* * .:: *: *: *. . .*:

hCoV-OC43 EELDQWFKNQTSVAPDLSL--DYINVTFLDLQVEMNRLQEAIKVLNQSYINLKDIGTYEY 1292

hCoV-HKU1 SELSHWFKNQTSIAPNLTLNLHTINATFLDLYYEMNLIQESIKSLNNSYINLKDIGTYEM 1295

MERS-CoV DELDEFFKNVSTSIPNFG-SLTQINTTLLDLTYEMLSLQQVVKALNESYIDLKELGNYTY 1291

SARS-CoV-2 EELDKYFKNHTSPDVDLG-DISGINASVVNIQKEIDRLNEVAKNLNESLIDLQELGKYEQ 1208

SARS-CoV EELDKYFKNHTSPDVDLG-DISGINASVVNIQKEID-LNEVAKNLNESLIDLQELGKYEQ 1188

.**..:*** :: :: **.:.::: *: ::: * **:* *:*:::*.*

hCoV-OC43 YVKWPWYVWLLICLAGVAMLVLLFFICCCTGCGTSCFK--KCGGCCDDYTGYQELVIK-T 1349

hCoV-HKU1 YVKWPWYVWLLISFSFIIFLVLLFFICCCTGCGSACFS--KCHNCCDEYGGHHDFVIK-T 1352

MERS-CoV YNKWPWYIWLGFIAGLVALALCVFFILCCTGCGTNCMGKLKCNRCCDRYEEYDLEPHKVH 1351

SARS-CoV-2 YIKWPWYIWLGFIAGLIAIVMVTIMLCCMTSCCSCLKGCCSCGSCCKF-DEDDSEPVLKG 1267

SARS-CoV YIKWPWYVWLGFIAGLIAIVMVTILLCCMTSCCSCLKGACSCGSCCKF-DEDDSEPVLKG 1247

* *****:** : . : : : ::: * *.* : .* **. .

hCoV-OC43 SHDD-- 1353

hCoV-HKU1 SHDD-- 1356

MERS-CoV VH---- 1353

SARS-CoV-2 VKLHYT 1273

SARS-CoV VKLHYT 1253

:
